# Association of Head and Neck Cancers in Chronic Osteomyelitis: A National Retrospective Cohort Study: Erratum

**DOI:** 10.1097/01.md.0000483410.54264.a4

**Published:** 2016-05-13

In the articles “Association of Head and Neck Cancers in Chronic Osteomyelitis: A National Retrospective Cohort Study”,^1^ Dr. Ming-Chia Lin's affiliation was not reported in full. Dr. Lin is affiliated with the E-Da Hospital. The articles have since been corrected online.
